# A Sensitive Approach to Studying ASDs: Teasing Out Relationships between Autism and Maternal Smoking

**DOI:** 10.1289/ehp.120-a285b

**Published:** 2012-07-02

**Authors:** Tanya Tillett

**Affiliations:** Tanya Tillett, MA, of Durham, NC, is a staff writer/editor for *EHP*. She has been on the *EHP* staff since 2000 and has represented the journal at national and international conferences.

Both genetic and environmental factors have been implicated in autism spectrum disorders (ASDs), which affect an estimated 1 in 88 children. One such environmental factor, prenatal exposure to tobacco smoke via maternal smoking, has been associated with ASDs in some studies but not others. A new study reports evidence of a positive association between maternal smoking during pregnancy and higher-functioning ASD subtypes [*EHP* 120(7):1042–1048; Kalkbrenner et al.].

The authors collected information on maternal smoking and other factors from the birth certificates for 633,989 children born in 1992, 1994, 1996, and 1998 in 11 U.S. states. They linked these data with surveillance data from the U.S. Centers for Disease Control and Prevention’s Autism and Developmental Disabilities Monitoring network and identified 3,315 of the children who were subsequently diagnosed with an ASD by age 8 years.

About 13% of all the mothers smoked during pregnancy, compared with about 11% of mothers with children diagnosed with an ASD. Maternal smoking has been associated with both lower education and reduced access to health care, factors that might increase the likelihood that ASDs go undiagnosed among children of women who smoked during pregnancy. When the authors corrected for this potential bias using outcome misclassification sensitivity analyses, a weak positive association emerged between maternal smoking and cases classified as “ASD not otherwise specified,” which were assumed to be higher-functioning ASDs such as Asperger’s disorder. The association was not found for lower-functioning (that is, more severe) ASDs.

The authors write that their findings concerning ASD subgroups should be interpreted with caution because the accuracy of subgroup classification may have varied depending upon mothers’ access to evaluation services, and because it was not bas ed on direct clinical observation. They also note that positive associations may reflect the presence in higher-functioning subgroups of children with co-occurring disorders (such as attention deficit/hyperactivity disorder) that can be affected by nicotine exposure.

Strengths of the study include the large sample size, the population-based design with standardized identification of ASD cases, and the use of sensitivity analyses to evaluate potential sources of bias. The authors conclude that the observed association between maternal smoking during pregnancy and higher-functioning ASDs warrants further research.

